# The effect of trust, IT knowledge, and entrepreneur’s innovativeness to embrace or shun the internet of things

**DOI:** 10.3389/fpsyg.2022.1035015

**Published:** 2022-11-24

**Authors:** Ahmad Abushakra, Davoud Nikbin, Ammar Odeh, Rasha Abdulwahab

**Affiliations:** ^1^Business Information Technology Department, Princess Sumaya University for Technology, Amman, Jordan; ^2^School of Business and Law, University of Brighton, Brighton, United Kingdom; ^3^Computer Science Department, Princess Sumaya University for Technology, Amman, Jordan; ^4^Department of Information Technology, University of Technology and Applied Sciences, Sohar, Oman

**Keywords:** entrepreneurs, internet of things, entrepreneurship, structural equation modeling, deliberate practices

## Abstract

This study examines critical factors influencing Omani entrepreneurs’ adoption of the internet of things (IoT) by expanding the constructs at the unified theory of acceptance and use of technology (UTAUT2) with entrepreneurs’ innovativeness, IT knowledge (ITK), and trust. A cross-sectional survey questionnaire was used to collect data from 158 entrepreneurs in Oman. Data were analyzed through the structural equation modeling technique using SmartPLS. The results indicated that performance expectancy, habit, social influence, trust (TR), ITK, and entrepreneurs’ innovativeness (PI) significantly affect Omani entrepreneurs’ intention to adopt IoT. Nonetheless, the results show that there is no significant relationship between hedonic motivation, effort expectancy, price value, and facilitating conditions to adopt IoT. This study contributes to previous literature by incorporating entrepreneurs’ innovativeness, ITK, and trust into UTAUT2. Furthermore, this study was conducted in a Middle Eastern country with solid support from the government for entrepreneurs; also, there is a gap in such studies in this area. This study helps practitioners in the field better understand how to influence entrepreneurs, push them toward using IoT applications further, and encourage non-users to start using them.

## Introduction

Since the use of information and telecommunication has increased exponentially, Internet technology has become increasingly relevant in all areas of business operations (Barba-Sanchez et al., 2018; Qi and Ismail, 2019). Among these technologies is the internet of things (IoT). The IoT provides real-time connectivity to numerous entities and objects (Sievers et al., 2021). Thanks to IoT providing real-time connectivity with every entity and object, recognizable objects can communicate with anyone and be accessed from anywhere (Gubbi et al., 2013). Despite the fact that IoT technology has been widely used in many parts of the world, including some Asian and Middle Eastern countries, it is considered primitive and undeveloped in Oman. In the last few years, IoT technologies have gained wide acceptance across a variety of industries, such as healthcare (Karahoca et al., 2018), logistics (Dong et al., 2017), supply chain management (Hsu and Lin, 2016), retail, agriculture, transportation, and public services (Dong et al., 2017). With the increased use of this new technology in a number of industries, the adoption of IoT also poses new, challenging questions in terms of how it is perceived by individuals, such as entrepreneurs (Li et al., 2020).

The literature has proposed various models to study users’ intentions to adopt technology. Several popular models include TAM, TRA, TPB, UTAUT, and UTAUT2. UTAUT was introduced by Venkatesh et al. (2003) in a study comparing a variety of technologies adoption models. Four constructs were included in this model: effort expectancy (EE), performance expectancy (PE), facilitating conditions (FC), and social influence (SI). All competing models were compared with this model and it was shown that it outperformed them all. UTAUT was later modified by Venkatesh et al. (2012) by incorporating a few more variables such as habit (HT), hedonic motivation (HM), and price value (PV) to create unified theory of acceptance and use of technology (UTAUT2). In spite of the wide use of UTAUT/UTAUT2 in Western countries, researchers have disagreed with its results because it reveals different biases across countries and contexts (Dwivedi et al., 2011; Teo et al., 2015).

This research aims to fill in the abovementioned gaps and examine how trust, IT knowledge (ITK), and entrepreneurs’ innovativeness influence the adoption of the IoT. By doing so, this research contributes in a number of ways to previous literature on technology adoption. Specifically, this research examines the adoption of IoT from the perspective of individual entrepreneurs, which has not been examined in previous literature. Secondly, this study extends UTAUT2 by considering constructs such as individual entrepreneurs’ innovativeness, their ITK, and trust, which are all highly relevant in the context of Oman. In addition, this research was carried out in an area of the Middle East where empirical studies have not been conducted. This research has the potential to enhance UTAUT2’s applicability and sturdiness.

## Theoretical framework and hypothesis development

### The UTAUT

Technology adoption has been extensively examined in the literature and has attracted research interest. There have been a number of theories, including the innovation diffusion theory (IDT; Rogers, 1995), theory of planned behavior (TPB; Ajzen, 1991), theory of reasoned action (TRA; Fishbein and Ajzen, 1975), and technology acceptance model (TAM; Davis, 1989), that have been developed and implemented to clarify the intention to use the latest technologies. Although, as mentioned above, these as well as all other technology adoption theories are prone to limitations, Venkatesh et al., 2003, therefore introduced UTAUT by synthesizing eight technological adoption models (TAM, TRA, TPB, IDT, TAM, the Motivational Model (Davis et al., 1992), the Model of PC Utilization (Thompson et al., 1991), and Innovation and Social Cognitive Theory (Compeau and Higgins, 1995)). The model was designed to overcome the limitations of other models, incorporating four primary constructs: EE, PE, SI, and FC (Venkatesh et al., 2003).

**Figure 1:**
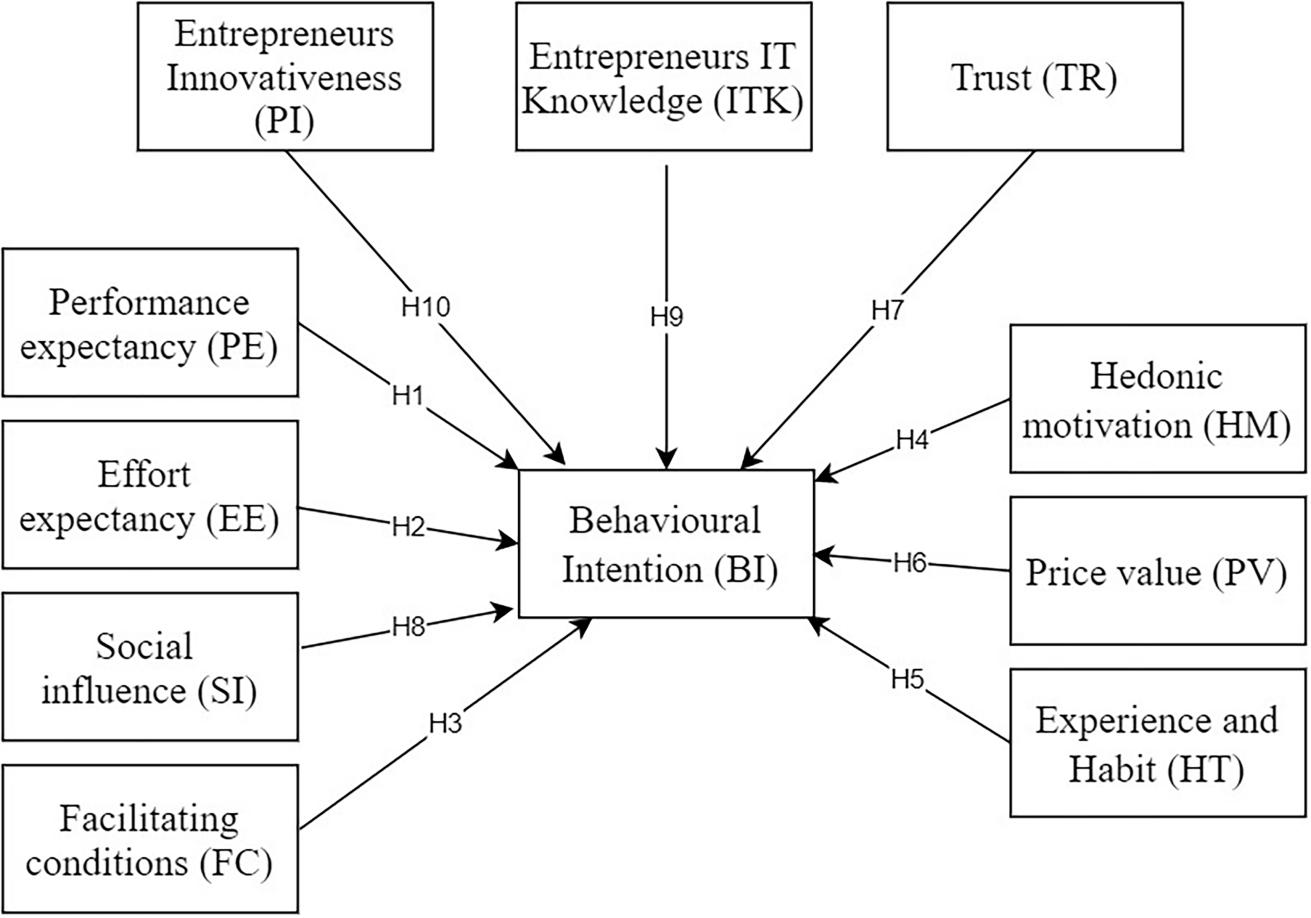
Research model.

In a similar manner to other acceptance and adoption models, it was also found that UTAUTs could be useful primarily for organizational contexts rather than individual consumer contexts (Negahban and Chung, 2014). The UTAUT has been revised by Venkatesh et al. (2012) in order to explore voluntary usage and better adapt it to the consumer use framework by incorporating factors such as HM, PV, and HT. This model has been used in several areas, including e-learning (El-Masri and Tarhini, 2017), online shopping (Tarhini et al., 2019), e-services (Fakhoury and Aubert, 2017), smartphones (Ameen et al., 2018), mobile banking (Alalwan et al., 2017; Baptista and Oliveira, 2017; Merhi et al., 2019), and Internet banking (Alalwan et al., 2015), as a comprehensive tool to understand consumer or organization intentions to adopt a technology. This study, therefore, extended UTAUT2 by taking into account entrepreneurs’ innovation, ITK, and trust. Below is a brief explanation of the constructs involved within our extended model that follows (Figure 1).

PE measures the level of benefits users will gain by utilizing technology to accomplish specific tasks and activities (Venkatesh et al., 2003; Brown and Venkatesh, 2005). A similar relationship exists between PE, relative advantage in Innovation Diffusion Model, and perceived usefulness in TAM. UTAUT (Venkatesh et al., 2003) and UTAUT2 (Venkatesh et al., 2012) have shown that this construct strongly influences behavioral intentions. Several past studies indicate that PE is also strongly linked to behavioral intentions in many contexts, such as massive online courses (Tseng et al., 2019), mobile apps for restaurants (Palau-Saumell et al., 2019), and online learning (Arpaci, 2015). When entrepreneurs believe that the IoT is valuable and can bring benefit to their operations and processes, they are more likely to adopt it and our research hypothesizes that:

*H1*: PE has a positive influence on entrepreneurs’ intention to adopt IoT.

The concept of effort expectation (EE) refers to the simplicity with which a technology or system is used (Venkatesh et al., 2003; Brown and Venkatesh, 2005). The construct is similar in meaning to complexity (Thompson et al., 1991) and perceived ease of use (Davis, 1989) in other models of technology acceptance. Among inexperienced users, EE significantly impacts behavioral intentions but not those of experienced users, according to (Venkatesh et al., 2003). Studies have previously linked EE to intention to adopt the IoT (Al-Momani et al., 2016), e-learning (Tarhini et al., 2017), and mobile commerce (Eneizan et al., 2019). When entrepreneurs find IoT easy to use, we expect they will be more likely to use it in their businesses, and based on that, we hypothesize that:

*H2*: EE has a positive influence on entrepreneurs’ intention to adopt IoT.

The FC describe how consumers perceive the availability of resources and support to perform a behavior (Venkatesh et al., 2003; Brown and Venkatesh, 2005). In general, this construct is taken from the FC Utilization model in (Thompson et al., 1991) and included as part of UTAUT in (Venkatesh et al., 2003). This construct is similar to the constructs such as compatibility (Moore and Benbasat, 1991) and perceived behavioral control (Ajzen, 1991). In the case of voluntary settings, this construct has been shown to be associated with behavioral intentions across previous literature. For example, a study conducted by El-Masri and Tarhini (2017), found a link between the FC that influence the adoption of online learning. This relationship has also been demonstrated in other contexts, such as the development of restaurant apps (Palau-Saumell et al., 2019) and the marketing of m-commerce (Eneizan et al., 2019). Using the available information, we hypothesize that external resources will affect the entrepreneur’s intention to adopt IoT. This is implied through the following hypothesis:

*H*3: FC has a positive influence on entrepreneurs’ intention to adopt IoT.

“HM” is a term used to describe an individual’s level of pleasure and entertainment caused by technology (Venkatesh et al., 2012; Tarhini et al., 2017). In fact, it was one of the three new components that were incorporated into UTAUT to form UTAUT2. It has previously been shown that in the literature of the information technology field as well as in consumer behavior, the role of HM (e.g., enjoyment, playfulness, entertaining) influences people’s intention to use ICTs (e.g., Childers et al., 2001; Brown and Venkatesh, 2005). Technology is more likely to be used by individuals when they find it entertaining and when it consists of creative tools and functions (Alalwan et al., 2017). According to research published by Lowry et al. (2012), intrinsic motivations such as curiosity and joy significantly impacted technology adoption. A variety of studies have found an association between HM and an individual’s intention to adopt technology in contexts such as Internet banking (Arenas Gaitán et al., 2015), social networking website adoption (Järvinen et al., 2016), online shopping (An et al., 2016), mobile technology (Huang and Kao, 2015), and online learning (Tarhini et al., 2016). Taking all of these studies into account, our hypothesis is that:

*H4*: HM has a positive influence on entrepreneurs’ intention to adopt IoT.

The HT can be described as the individual’s perception of doing things regularly (Tarhini et al., 2017), i.e., what he does daily. Additionally, Venkatesh et al. (2012) defined HT as a “perceptual construct or mental model that is based on previous experiences.” HT is all about automaticity, where individuals have a tendency to engage in a particular behavior because of what they have learned in the past (Limayem et al., 2007). In the past, a number of studies have indicated that HT plays a significant role in technology adoption, such as those conducted by Albugami and Bellaaj (2014), in e-services, by Slade et al. (2015) in mobile payments, and by Moura et al. (2017), in internet services. In light of the above, the following hypothesis can be formulated:

*H5*: HT has a positive influence on entrepreneurs’ intention to adopt IoT.

In typical professional practice, PV is defined as the individual’s perception of the price paid for goods and services and the benefits of having those goods and services (Venkatesh et al., 2012; Tarhini et al., 2017). However, in this study, PV refers to the client’s rational assessment of the benefits and the cost of using a good or service. It is a common belief that when individuals decide to adopt technology, they compare the utilitarian benefits they have gained from using the technology with the monetary cost it might have cost them (Venkatesh et al., 2012). It has been found that individuals are more likely to adopt a technology that has a higher PV if they find that technology to be more valuable (Dhiman et al., 2019). There has been previous research in this area that has revealed PV has an effect on the intent to adopt new technologies in different contexts. This is the case, for example, most obvious when looking at TV streaming (Haryoto, 2015), mobile banking (Mahfuz et al., 2020), and music streaming services (Helkkula, 2016). Based on these findings, the following hypothesis can be developed:

*H6*: PV has a positive influence on entrepreneurs’ intention to adopt IoT.

Trust (TR) is defined as “individual willingness to trust based on belief in capability, benevolence, and integrity” (Gefen, 2000). It has been emphasized in the past literature that trust is a key element to the use of the Internet and technological innovation. According to Tarhini et al. (2016), people’s decisions regarding the adoption or non-adoption of a new technology may be subordinated to a matter of trust and security with regard to the use of the technology. Our study found that entrepreneurs’ intention to use the IoT is determined by their confidence in this technology. So, we believe that the use of IoT will be more likely among entrepreneurs if they have confidence in the technology. In light of this, the following hypothesis is proposed:

*H7*: TR has a positive influence on entrepreneurs’ intention to adopt IoT.

According to Brown and Venkatesh (2005) and Venkatesh et al. (2003), SI describes an individual’s perception of the level of other significant people’s use of newly developed technologies or systems. Similar to the subjective norm outlined in the TRA, this norm was taken from TRA initially (Cooper et al., 2001), and it was then added to the UTAUT, and eventually to UTAUT2. A social setting facilitates the process of acceptance by a group of people and the belief that these norms should be followed by individuals. A number of studies supporting the positive influence of SI on behavioral intentions have been conducted, such as that found in e-learning (El-Masri and Tarhini, 2017), mobile banking (Eneizan et al., 2019), and mobile games (Ramírez-Correa et al., 2019). As a result of these observations, the following hypothesis was formulated:

*H8*: SI has a positive influence on entrepreneurs’ intention to adopt IoT.

The concept of ITK has been viewed from various perspectives. According to Group (2014), it refers to the ability of customers to use the applications on the Internet and the Internet itself, such as IoT services. In the context of employees’ perspectives, Zhu et al. (2003), define this as the employee’s knowledge of website development, Internet security, and other related technologies. Similarly, Sakarji et al. (2019), state that ITK refers to the technical expertise of the staff and their ability to use, interact, and retrieve information from information systems. As per Dimitrova and Chen (2006), individuals are more likely to adopt new technology if they are aware of it and understand how to utilize it effectively. This will reduce their levels of anxiety. Literature has proven the importance of information technology knowledge in technology adoption. Among others, one study (Han et al., 2014) on individuals’ intention to use third-party apps found that awareness of the technology significantly influences their decision to adopt that technology. Furthermore, it has been demonstrated that ITK can play an effective role in influencing the adoption of technology in Arab countries. In Jordan, for instance, Khasawneh et al. (2011), investigated the adoption of e-government services and found that computer and literacy significantly impact individuals’ intentions to adopt electronic services. Also, it is noteworthy that Al-Somali et al. (2015), found that the ITK of employees is one of the constructs that influence the individual’s intent to adopt new technology. As a consequence, we hypothesize that:

*H9*: ITK has a positive influence on entrepreneurs’ intention to adopt IoT.

It is commonly understood that entrepreneurial innovation (PI) refers to the process of developing or adopting ideas or new behaviors related to technology, product, service, system, or practice (Damanpour and Wischnevsky, 2006). The second definition of personal innovativeness is defined by (Rogers, 2010) as “the degree to which an individual is relatively early in adopting new ideas than are average other members of a system.” By this definition, it can be determined that an individual is high in personal innovativeness if he/she is open to fresh ideas and has an eagerness to realize that new possibilities exist. According to a study (Thong, 1999), the innovativeness of CEOs is associated with their intention to implement information systems in their businesses. In the same vein, in this study, we expect that the intention of entrepreneurs to adopt IoT depends upon their flexibility to be open to new ideas as well as their ability to be innovative. As a result, we hypothesized that:

*H10*: PI has a positive influence on entrepreneurs’ intention to adopt IoT.

## Methodology

### Procedure

Our study was conducted in the context of the adoption of the IoT in the country of Oman. In order to collect data, 158 Omani entrepreneurs were surveyed using a self-administered questionnaire. As the original survey was created in English, we used the back translation method in order to validate the survey. The original English survey was then translated into Arabic by a linguist, and another linguist was then hired to translate it back into Arabic. Both versions were compared for consistency. As recommended by Sekaran and Bougie (2011), before embarking on the survey, three experts and three academicians in the field reviewed it for validity regarding its content and face. We tailored items to suit our study context. We received comments on the questions, then assessed the validity of the items based on interviews with three experts to confirm their content. Moreover, the three academicians checked the content to ensure the content was comprehensive enough to represent all constructs. The survey questions were slightly modified based on comments received from experts, academicians, and participants of the pilot study to make the study more straightforward, not to create confusion, and to fit the context of our study. For the purposes of collecting data, the researchers attended the Injaz Oman Student Companies Exhibition. We have applied convenience sampling to collect the data from the respondents as they have been approached during the event and asked to participate in this survey. It is important to point out that participation in the survey was voluntary, and that all participants were informed as to what the purpose of this study was. We informed the participants that during the data collection process, they had the right to withdraw from completing the survey form at any time or for any reason.

### Measurement

In the current exploratory study, all questions for measuring the latent variable originate from previous literature related to UTAUT2. However, certain modifications were made to the questions to put them into the context of the current study. More specifically, five measuring items have been used in order to measure performance, five items to measure EE, and five to measure the price. Additionally, within each construct, four questions were used to measure other latent variables. We have adapted a number of the items related to the above-mentioned latent variables with minor modifications from Venkatesh et al. (2012), Tarhini et al. (2016), Teo (2009), and Venkatesh and Zhang (2010). There was no conflict between any of the questions used to measure the variables. All of the questions passed on a seven-point Likert scale ranging from 1 to 7, 1 representing “strongly disagree, “and 7 representing “strongly agree” (Table 1).

**Table 1:** Summary of construct with measurement items.

Construct	Corresponding items		Items sources
Performance expectancy (PE)	PE1	I find Internet of things (IoT) useful in my daily life.	Venkatesh et al. (2003)
	PE2	Using Internet of things (IoT) increases my chances of achieving tasks that are important to me	
	PE3	Using Internet of things (IoT) helps me accomplish tasks more quickly.	
	PE4	Using Internet of things (IoT) increases my productivity	
Effort expectancy (EE)	EE1	Learning how to use Internet of things (IoT) is easy for me.	Venkatesh et al. (2003)
	EE2	My interaction with Internet of things (IoT) is clear and understandable.	
	EE3	I find Internet of things (IoT) easy to use.	
	EE4	It is easy for me to become skillful at using Internet of things (IoT).	
Social influence (SI)	SI1	People who are important to me think that I should use Internet of things (IoT).	Venkatesh et al. (2003)
	SI2	People who influence my behavior think that I should use Internet of things (IoT).	
	SI3	My family/friends frequently use Internet of things IoT services.	
	SI4	People whose opinions that I value prefer that I use Internet of things (IoT).	
Facilitating conditions (FC)	FC1	I have the resources necessary to use Internet of things (IoT).	Ifinedo (2012), Venkatesh et al. (2012)
	FC2	I have the knowledge necessary to use Internet of things (IoT).	
	FC3	Internet of things (IoT) is compatible with other technologies I use.	
	FC4	I can get help from others when I have difficulties using Internet of things (IoT).	
Hedonic motivation (HM)	HM1	Using Internet of things (IoT) is fun.	Venkatesh et al. (2012)
	HM2	Internet of things (IoT) is enjoyable.	
	HM3	Using Internet of things (IoT) is entertaining	
Price value (PV)	PV1	Internet of things (IoT) is reasonably priced.	Sakarji et al. (2019)
	PV2	Internet of things (IoT) is good value for the money.	
	PV3	At the current price, Internet of things (IoT) provides good value.	
Experience and habit (EH)	HT1	The use of Internet of things (IoT) has become a habit for me.	Tu (2018)
	HT2	I am addicted to using Internet of things (IoT).	
	HT3	I must use Internet of things (IoT).	
IT knowledge (ITK)	ITK1	I have a good knowledge of the Internet and its applications	Al-Somali et al. (2015)
	ITK2	I would rate my understanding of information technology (IT) as very good compared to my peers.	
	ITK3	I have expert knowledge of information Technology (IT) and e-commerce technologies	
Personal innovativeness (PI)	PI1	If I heard about a new information technology, I would look for ways to experiment with it	Karaiskos et al. (2009)
	PI2	Among my peers, I am usually the first to try out new information Technologies.	
	PI3	In general, I am not hesitant to try out new information technologies.	
	PI3	I like to experiment with new information technologies	
Trust (TR)	TR1	I believe that Internet of things (IoT) is trustworthy.	Pavlou et al. (2003)
	TR2	I trust in Internet of things (IoT).	
	TR3	I do not doubt the honesty of Internet of things (IoT).	
	TR4	I feel assured that legal and technological structures adequately protect me from problems on Internet of things (IoT).	
	TR5	Even if not monitored, I would trust Internet of things (IoT) to do the job right.	
	TR6	Internet of things (IoT) has the ability to fulfil its task.	
Behavioral Intention (BI)	BI1	I intend to use Internet of things (IoT) in the future.	Alabdullah et al. (2020)
	BI2	I will always try to use the Internet of things (IoT) daily.	
	BI3	I plan to use Internet of things (IoT) in future.	
	BI4	I predict I would use Internet of things (IoT) in the future.	
	BI5	I intend to recommend my friends to use Internet of things (IoT) services in the future.	

### Data analysis

Data collected for this research were analyzed using partially least squares (PLS) and SmartPLS 2.0 (Sarstedt et al., 2021). For this reason, a PLS method was used in this study (Henseler et al., 2016), since it is highly suitable for small-to-medium samples. Each latent variable used in this study was treated as a reflective variable. There are two phases in the SmartPLS, namely the measurement model and structural model evaluation. With regard to the measurement model evaluation, we evaluated the internal consistency, reliability, convergent validity, and discriminant validity, whereas regarding the structural model evaluation, we assessed the path coefficients, their *p*-values, and whether the hypotheses were supported or not. The purpose of this approach was to verify both the validity and the reliability of the measurements before the structural relationships within the model were evaluated (Table 2).

**Table 2:** Demographics of respondents.

Variable	Description	Frequency	Percentage
Gender	Male	80	50.6
	Female	78	49.4
Age	18–25	112	70.9
	26–35	36	22.8
	36–45	9	5.7
	46 and Above	1	0.6
Marital status	Single	94	59.5
	Married	59	37.3
	Divorced	3	1.9
	Window	2	1.3
Education	High School	6	3.8
	Diploma	43	27.2
	Bachelor’s	109	69.0
	Master and above	0	0
Region	Ad Dakhiliyah	12	7.6
	Ad Dhahirah	26	16.5
	Al Batinah	70	44.3
	Al Buraimi	4	2.5
	Al Wusta	2	1.3
	Ash Sharqiyah	9	5.7
	Muscat	35	22.2

## Results

### Measurement model assessment

As part of the measurement model evaluation, the items are checked for reliability, internal consistency of the measures, and discriminant validity. In order to determine the reliability of the individual item, factor loadings of each construct on its corresponding construct were examined. Based on the findings of Hair et al. (2011), items with individual loadings of 0.6 and above can be accepted and kept for further analysis, while items with individual loadings of less than 0.6 can be removed. In this study, all items were examined separately and the reliability for each item was 0.6 or more, thus meeting the threshold of acceptance.

Ideally, the items that measure a latent construct should have a high level of internal consistency. In order to ensure that there is adequate internal consistency reliability, we need to make sure the composite reliability (CR) and Cronbach’s alpha are both 0.7 at least. All latent variables used in this study were found to comply with the internal consistency criteria, as the Cronbach alpha and CR values were all greater than 0.70. Additionally, the average variance extracted (AVE) was also checked to determine whether the variables fit the criteria for internal consistency. It was found that all latent variables measured in this research had a variability above 0.5, in line with the recommendation of Fornell and Larcker (1981). Accordingly, the findings of this study are significant in supporting internal consistency (Table 3).

**Table 3:** Convergent validity of the constructs.

Construct	Item	Factor loadings	Composite reliability (CR)	Average variance extracted (AVE)	Cronbach’s alpha
PE			0.932	0.774	0.903
	PE1	0.899			
	PE2	0.900			
	PE3	0.873			
	PE4	0.847			
EE			0.912	0.722	0.871
	EE1	0.862			
	EE2	0.892			
	EE3	0.839			
	EE4	0.803			
FC			0.921	0.745	0.884
	FC1	0.757			
	FC2	0.905			
	FC3	0.907			
	FC4	0.874			
HM			0.919	0.790	0.868
	HM1	0.862			
	HM2	0.919			
	HM3	0.885			
HT			0.909	0.770	0.850
	HT1	0.828			
	HT2	0.917			
	HT3	0.886			
PV			0.925	0.804	0.877
	PV1	0.842			
	PV2	0.925			
	PV3	0.920			
TR			0.917	0.688	0.886
	TR2	0.839			
	TR3	0.815			
	TR4	0.814			
	TR5	0.890			
	TR6	0.786			
SI			0.844	0.576	0.755
	SI1	0.729			
	SI2	0.782			
	SI3	0.809			
	SI4	0.712			
ITK			0.885	0.720	0.806
	ITK1	0.824			
	ITK2	0.889			
	ITK3	0.831			
PI			0.928	0.764	0.897
	PI1	0.836			
	PI2	0.861			
	PI3	0.913			
	PI4	0.885			
BI			0.896	0.633	0.855
	BI1	0.804			
	BI2	0.768			
	BI3	0.831			
	BI4	0.815			
	BI5	0.759			

Besides the above, discriminant validity was evaluated as the last assessment in the measurement model. The cross-loading of each indicator has been checked for validity and found that none of the indicators load higher than the other on opposing constructs (Hair et al., 2013). Moreover, the criterion of Fornell and Larcker (1981), was also used to compare the correlation between other constructs and the square root of the AVEs of the constructs discussed here. Interestingly, the results of the analysis confirmed that all diagonal values were significantly higher than the values in the corresponding rows and columns, indicating that each construct is different from the others in the model (Table 4).

**Table 4:** Discriminant validity of the constructs.

	PE	EE	FC	HM	HT	PV	TR	SI	ITK	PI	BI
PE	**0.880**										
EE	0.608	**0.850**									
FC	0.468	0.515	**0.863**								
HM	0.387	0.391	0.483	**0.889**							
HT	0.447	0.463	0.418	0.418	**0.887**						
PV	0.366	0.440	0.567	0.573	0.544	**0.896**					
TR	0.504	0.574	0.524	0.460	0.600	0.509	**0.829**				
SI	0.589	0.623	0.618	0.473	0.538	0.505	0.625	**0.759**			
ITK	0.541	0.487	0.428	0.474	0.491	0.460	0.567	0.533	**0.848**		
PI	0.448	0.463	0.499	0.422	0.502	0.493	0.551	0.537	0.571	**0.874**	
BI	0.596	0.560	0.595	0.540	0.649	0.586	0.706	0.691	0.660	0.722	**0.924**

The diagonal (in Bold) represents the square root of average variance extracted (AVE) and the sub-diagonals, the inter-construct correlation values.

### Structural model assessment

The second step of PLS-SEM is an assessment of the structural model. We estimated the path coefficients using the PLS algorithm to test the structural model’s effectiveness, and the statistical significance was calculated using bootstrapping with 500 replications, as suggested by Hair et al. (2016). Considering the results of the study, it appears that most of the hypotheses are supported (Table 5). The results indicated that PE, HT, TR, SI, ITK, and PI have a significant relationship with BI, while EE, FC, HM, and PV do not. Thus, PE, HT, TR, SI, ITK, and PI have a meaningful relationship with BI, as hypothesized and as expected. Due to this, *H*1, *H*5, *H*7, *H*8, *H*9, and *H*10 were all supported. However, *H*2, *H*3, *H*4, and *H*6 were not.

**Table 5:** Hypothesis testing.

Hypothesis	Relationship	Path coefficient	Std. Dev.	*t*-Values	Decision
H1	PE => BI	0.111	0.052	2.131	Supported
H2	EE => BI	−0.043	0.057	0.754	Not Supported
H3	FC => BI	0.067	0.057	1.181	Not Supported
H4	HM => BI	0.06	0.060	1.004	Not Supported
H5	HT => BI	0.15	0.060	2.586	Supported
H6	PV => BI	0.047	0.064	0.737	Not Supported
H7	TR => BI	0.176	0.070	2.53	Supported
H8	SI => BI	0.146	0.060	2.433	Supported
H9	ITK => BI	0.127	0.057	2.227	Supported
H10	PI => BI	0.287	0.050	5.716	Supported

*p < 0.05, **p < 0.01, ***p < 0.001 (one tail) 0.1.65*, 1.96**, 2.57***.

## Discussion

Results of this study showed that PE, HT, TR, SI, ITK, and PI positively affected intentions to adopt the IoT. In addition, EE, FC, HM, and PV were not considered in terms of their effects on the intention to adopt IoT services.

More specifically, this study found that the PE of the IoT positively affects Omani entrepreneurs’ intention to adopt it. It implies that entrepreneurs may adopt IoT if they feel that it can be helpful for productivity and task completion in their lives. This is in accordance with previous research findings that found a positive and significant relationship between the two (Tarhini et al., 2016, 2017; El-Masri and Tarhini, 2017). The results of this research work also revealed that contrary to what was hypothesized, there is no significant relationship between EE and entrepreneurs’ intentions to adopt IoT. Our results contradict those in the previous literature, in which most studies found a significant relationship between EE and intention to adopt a new technology (Tarhini et al., 2016, 2017; El-Masri and Tarhini, 2017). This insignificant relationship may be due to the fact that most Omani entrepreneurs are still unfamiliar with the IoT and lack information technology literacy and skills. Similar to this study, few previous research studies also found no significant relationship between them (Hossain et al., 2019).

As well, this research found no significant relationship between FC and intention to adopt IoT, contradicting most previous studies (El-Masri and Tarhini, 2017; Hossain et al., 2019). It is believed that the insignificant relationship between enabling conditions and intent to adopt IoT originates from a lack of resources, knowledge, and expertise, all of which can adversely affect this relationship. Accordingly, hypothesis 4 was rejected because HM was not significantly correlated with entrepreneurs’ intention to adopt IoT. The reason for this may be the fact that entrepreneurs did not perceive technology as fun, enjoyable, and entertaining. Consequently, cause of that fact, they do not have any intention to adopt it. In fact, this result does not align with the findings of previous studies like (El-Masri and Tarhini, 2017).

The study also found a positive correlation between HT and behavioral intentions, supporting previous research related to new technology adoption (Kalamatianou and Malamateniou, 2017). Once people become accustomed to using the IoT and get addicted to it as a new technology, they will be more likely to adopt and use it in their daily operations. Furthermore, contrary to what was anticipated, this study found no significant relationship between PV and intention to adopt IoT. Similar findings were reported in a previous study Tarhini et al. (2017). This insignificant relationship may result from the fact that Omani entrepreneurs may perceive Internet services provided by service providers as expensive and inefficient and this may prevent them from adopting the IoT in their day-to-day operations. Moreover, SI was confirmed to be one of the factors that influence an entrepreneur’s decision to adopt IoT. Therefore, when entrepreneurs compare themselves with peers and role models who use or recommend the technology, they are more likely to adopt it themselves, which confirms the findings of previous studies (Tarhini et al., 2016; El-Masri and Tarhini, 2017; Kalamatianou and Malamateniou, 2017).

**Figure 2:**
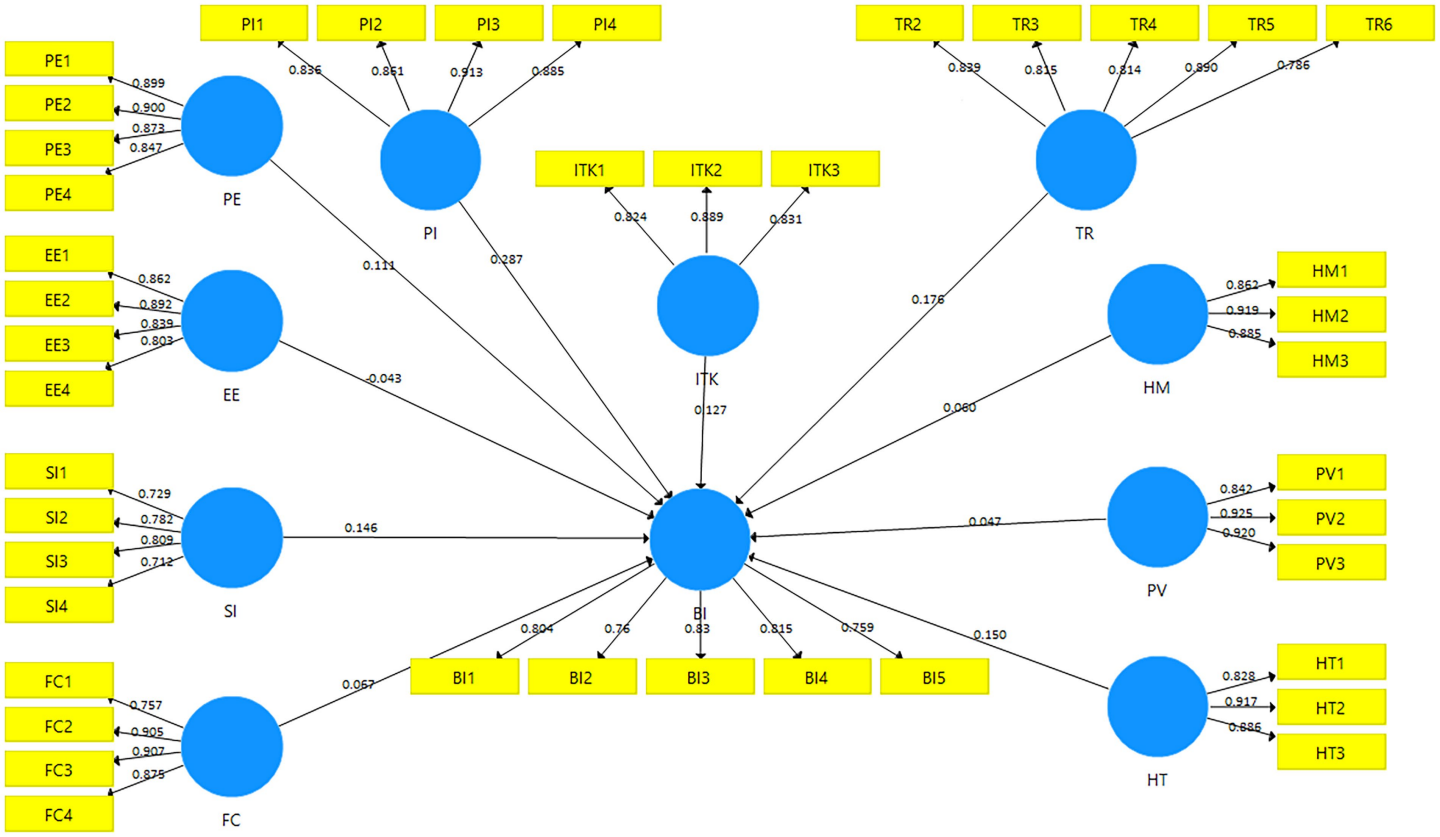
Structural model.

Further to the above, our findings confirmed the positive effects of ITK on the intention of Omani entrepreneurs to adopt IoT. Based on this result, the truth is that entrepreneurs who are familiar with IT, its applications, and e-commerce technologies will be more likely to adopt IoT technologies in their operations. We have observed similar conclusions in other previous studies conducted in the Arab region. As an example, a recent study by Khasawneh et al. (2011) found that factors related to computer literacy and the use of technology by Jordanians play a major role in their acceptance and adoption of this type of technology (electronic government services). In addition, our results have confirmed our hypotheses that the perspective of entrepreneurial innovativeness positively affects entrepreneurial intentions to adopt IoT. In this way, it becomes clear that entrepreneurs’ excitement and willingness to try out new technologies clearly affects their intention to use those technologies to streamline their operation in a more efficient way (Figure 2).

## Implications

Both theoretical aspect and practical of the work have been addressed. As a theoretical contribution, this study extends UTAUT2 knowledge, then tests it empirically in a developing country, such as Oman, to increase existing knowledge of technology acceptance and adoption. By extending UTAUT2 with trust, ITK, and entrepreneurial innovativeness, we built a conceptual model to better analyze entrepreneurs’ motivation to adopt the IoT. There are fewer studies that apply UTAUT2 in entrepreneurial research since most of the research implementing UTAUT2 has been conducted in other settings, including e-learning, e-services, e-commerce, etc. The study also addresses the call from Venkatesh et al. (2012) for research and testing of UTAUT2 across non-western and non-western cultures in order to enhance validity and generalizability. We have made a significant theoretical contribution to this study by considering ITK as an influential factor affecting behavioral intention in an Arab country such as Oman. The existence of this specific factor has been highlighted in previous research as a significant factor affecting technology adoption in Arab countries. However, this factor has not been explored in a study of UTAUT2. Moreover, it has never been investigated in relation to entrepreneurs’ intention to adopt a new technology like IoT. Study findings validated the importance of trust, ITK, and entrepreneurial innovation in a business’s intention to adopt IoT.

As well as providing important theoretical input, our study’s results also provide important implications for practitioners. In general, the results of this research are very helpful to IoT service providers as they can better understand entrepreneurs’ opinions on important factors influencing IoT adoption. This information will allow IoT developers to understand better what can be done to improve IoT applications. As users of IoT services, the entrepreneurial community can also better understand the reasons behind their adoption of IoT services. The results of our study showed that Omani entrepreneurs’ intention to adopt IoT is positively and significantly influenced by their experiences with performance expectations. The result clearly indicates that entrepreneurs are concerned about the quality and availability of IoT services. It is therefore recommended that first and foremost, IoT developers should ensure the quality of the products or services they provide to entrepreneurs. Developing smart objects for consumers (entrepreneurs) would require embedding sensors and communication systems. This is to ensure that smart devices offer something superior to non-IoT devices. As well, SI plays a very significant role in the adoption of IoT by Omani entrepreneurs. It seems that policymakers should employ SI to facilitate the emergence of entrepreneurs interested in implementing the IoT into their operations. In addition to that, IoT service providers can embrace word-of-mouth marketing and mass advertising to achieve a perception of critical mass and attract a large number of entrepreneurs. IoT service providers may find it useful to enlist entrepreneur users of IoT through social media platforms to share their experiences with smart technologies and how they save money through adopting them. This may attract even more entrepreneurs to use IoT. As an added benefit, our findings confirm that the effects of HTs on the intention to adopt IoT are stronger than that of technological advances alone. Therefore, IoT providers should emphasize the benefits of using this technology and help entrepreneurial companies implement technology in their operations to use it as an aspect of operation. As a result, if entrepreneurs follow this simple process, IoT may become ingrained in their minds and, therefore, they will likely utilize this technology.

As a consequence, the results of this study also demonstrated that trust plays a substantial role in an entrepreneur’s intention to adopt IoT. Hence, policymakers should pay close attention to the importance of establishing trust among entrepreneurs through their marketing strategies, which communicate the reliability of the IoT. The findings also confirmed that personal innovativeness had a significant effect on the intention to adopt IoT, supporting the idea that the providers of IoT services may need to provide incentives or triggers to attract those with high levels of personal innovativeness to use this technology. In addition to this, IoT marketers should also employ IoT pioneers and reward them for their contributions to attracting potential users *via* social networking sites to utilize this technology. It has also been found that ITK is a significant predictor of IoT adoption, which suggests that IoT providers should provide all the necessary support and training to entrepreneurs in order to encourage their intention to adopt IoT. The overall goal of this study (IoT developers and experts) was to better understand what entrepreneurs care about when it comes to such applications (IoT) and to discover what such entrepreneurs expect from them.

## Limitations and future research

As with any other type of study, this one too has its limitations that should be addressed in future studies. It is worth noting that the study in question concentrated on Omani entrepreneurs alone and that the results may not be generalizable to entrepreneurs in other countries. Therefore, future research might consider doing a replication in other countries or a cross-cultural study as the findings of our study were country-specific (Oman). This is the case because behavior differs in different countries due to a multitude of factors, including beliefs, social norms, culture, levels of adoption of new technologies, etc. A second concern is that the data for this study were collected by convenience sampling, so the sample may not necessarily reflect the entire population. Other sampling techniques may be considered to have a more accurate representation of the population in the future. The third limitation of this study is that data were collected through a survey and a cross-sectional approach, whereby using a survey may limit one’s ability to get deeper responses from respondents. Future research may likely adopt other approaches, such as interviews and qualitative and longitudinal approaches to understand entrepreneurs’ intentions better to adopt IoT. Finally, continuing research could explore the inclusion of other variables, such as demographic attributes, and personality (Antoncic et al., 2015, 2018; Peljko and Antončič, 2022) into our proposed study framework in the future.

## Data availability statement

The raw data supporting the conclusions of this article will be made available by the authors, without undue reservation.

## Author contributions

All authors listed have made a substantial, direct, and intellectual contribution to the work and approved it for publication.

## Conflict of interest

The authors declare that the research was conducted in the absence of any commercial or financial relationships that could be construed as a potential conflict of interest.

## Publisher’s note

All claims expressed in this article are solely those of the authors and do not necessarily represent those of their affiliated organizations, or those of the publisher, the editors and the reviewers. Any product that may be evaluated in this article, or claim that may be made by its manufacturer, is not guaranteed or endorsed by the publisher.

## References

[ref1] Ajzen I. (1991). The theory of planned behavior. Organizational behavior and decision processes. University of Massachusetts at Amherst: Academic Press. Inc.

[ref2] Alabdullah J. H. Van Lunen B. L. Claiborne D. M. Daniel S. J. Yen C. J. Gustin T. S. (2020). Application of the unified theory of acceptance and use of technology model to predict dental students’ behavioral intention to use teledentistry. J. Dent. Educ. 84, 1262–1269. doi: 10.1002/jdd.12304, PMID: 32705688

[ref3] Alalwan A. A. Dwivedi Y. K. Rana N. P. (2017). Factors influencing adoption of mobile banking by Jordanian bank customers: extending UTAUT2 with trust. Int. J. Inf. Manag. 37, 99–110. doi: 10.1016/j.ijinfomgt.2017.01.002

[ref4] Alalwan A. A. Dwivedi Y. K. Rana N. P. Lal B. Williams M. D. (2015). Consumer adoption of internet banking in Jordan: examining the role of hedonic motivation, habit, self-efficacy and trust. J. Financ. Serv. Mark. 20, 145–157. doi: 10.1057/fsm.2015.5

[ref5] Albugami M. Bellaaj M. (2014). The continued use of internet banking–combining UTAUT2 theory and service quality model. J. Glob. Manage. Res. 10, 11–28.

[ref6] Al-Momani A. M. Mahmoud M. A. Ahmad M. S. (2016). Modeling the adoption of internet of things services: a conceptual framework. Int. J. Appl. Res. 2, 361–367.

[ref7] Al-Somali S. A. Gholami R. Clegg B. (2015). A stage-oriented model (SOM) for e-commerce adoption: a study of Saudi Arabian organisations. J. Manuf. Technol. Manag. 26, 2–35. doi: 10.1108/JMTM-03-2013-0019

[ref8] Ameen N. Willis R. Shah M. H. (2018). An examination of the gender gap in smartphone adoption and use in Arab countries: a cross-national study. Comput. Hum. Behav. 89, 148–162. doi: 10.1016/j.chb.2018.07.045

[ref9] An L. Han Y. Tong L. (2016). Study on the Factors of Online Shopping Intention for Fresh Agricultural Products Based on UTAUT2. Paper Presented at the the 2nd Information Technology and Mechatronics Engineering Conference (ITOEC 2016).

[ref10] Antoncic J. A. Antoncic B. Grum D. K. Ruzzier M. (2018). The big five personality of the SME manager and their company’s performance. J. Dev. Entrep. 23:1850021. doi: 10.1142/S1084946718500218

[ref11] Antoncic B. Bratkovic Kregar T. Singh G. DeNoble A. F. (2015). The big five personality–entrepreneurship relationship: evidence from Slovenia. J. Small Bus. Manag. 53, 819–841. doi: 10.1111/jsbm.12089

[ref12] Arenas Gaitán J. Peral Peral B. Ramón Jerónimo M. (2015). Elderly and internet banking: An application of UTAUT2. J. Internet Bank. Commer. 20, 1–23.

[ref13] Arpaci I. (2015). A comparative study of the effects of cultural differences on the adoption of mobile learning. Br. J. Educ. Technol. 46, 699–712. doi: 10.1111/bjet.12160

[ref14] Baptista G. Oliveira T. (2017). Why so serious? Gamification impact in the acceptance of mobile banking services. Internet Res. 27, 118–139. doi: 10.1108/IntR-10-2015-0295

[ref15] Barba-Sanchez V. Calderón-Milán M. J. Atienza-Sahuquillo C. (2018). A study of the value of ICT in improving corporate performance: a corporate competitiveness view. Technol. Econ. Dev. Econ. 24, 1388–1407. doi: 10.3846/tede.2018.3114

[ref16] Brown S. A. Venkatesh V. (2005). A model of adoption of technology in the household: a baseline model test and extension incorporating household life cycle. Manag. Inf. Syst. Q. 29:399. doi: 10.2307/25148690

[ref17] Childers T. L. Carr C. L. Peck J. Carson S. (2001). Hedonic and utilitarian motivations for online retail shopping behavior. J. Retail. 77, 511–535. doi: 10.1016/S0022-4359(01)00056-2

[ref18] Compeau D. R. Higgins C. A. (1995). Application of social cognitive theory to training for computer skills. Inf. Syst. Res. 6, 118–143. doi: 10.1287/isre.6.2.118

[ref19] Cooper J. Kelly K. A. Weaver K. (2001). “Attitudes, norms, and social groups,” in Blackwell Handbook of Social Psychology: Group Processes. eds. M. A. Hogg and S. Tindale (wiley), 259–282.

[ref20] Damanpour F. Wischnevsky J. D. (2006). Research on innovation in organizations: distinguishing innovation-generating from innovation-adopting organizations. J. Eng. Technol. Manag. 23, 269–291. doi: 10.1016/j.jengtecman.2006.08.002

[ref21] Davis F. D. (1989). Perceived usefulness, perceived ease of use, and user acceptance of information technology. MIS Q. 13, 319–340. doi: 10.2307/249008

[ref22] Davis F. D. Bagozzi R. P. Warshaw P. R. (1992). Extrinsic and intrinsic motivation to use computers in the workplace 1. J. Appl. Soc. Psychol. 22, 1111–1132. doi: 10.1111/j.1559-1816.1992.tb00945.x

[ref23] Dhiman N. Arora N. Dogra N. Gupta A. (2019). Consumer adoption of smartphone fitness apps: an extended UTAUT2 perspective. J. Indian Bus. Res. 12, 363–388. doi: 10.1108/JIBR-05-2018-0158

[ref24] Dimitrova D. V. Chen Y.-C. (2006). Profiling the adopters of e-government information and services: the influence of psychological characteristics, civic mindedness, and information channels. Soc. Sci. Comput. Rev. 24, 172–188. doi: 10.1177/0894439305281517

[ref25] Dong X. Chang Y. Wang Y. Yan J. (2017). Understanding usage of internet of things (IOT) systems in China: cognitive experience and affect experience as moderator. Inf. Technol. People 30, 117–138. doi: 10.1108/ITP-11-2015-0272

[ref26] Dwivedi Y. K. Rana N. P. Chen H. Williams M. D. (2011). A Meta-Analysis of the Unified Theory of Acceptance and Use of Technology (UTAUT). Paper Presented at the IFIP International Working Conference on Governance and Sustainability in Information Systems-Managing the Transfer and Diffusion of It.

[ref27] El-Masri M. Tarhini A. (2017). Factors affecting the adoption of e-learning systems in Qatar and USA: extending the unified theory of acceptance and use of technology 2 (UTAUT2). Educ. Technol. Res. Dev. 65, 743–763. doi: 10.1007/s11423-016-9508-8

[ref28] Eneizan B. Mohammed A. G. Alnoor A. Alabboodi A. S. Enaizan O. (2019). Customer acceptance of mobile marketing in Jordan: An extended UTAUT2 model with trust and risk factors. Int. J. Eng. Bus. Manag. 11, 1–10. doi: 10.1177/1847979019889484

[ref29] Fakhoury R. Aubert B. (2017). The impact of initial learning experience on digital services usage diffusion: a field study of e-services in Lebanon. Int. J. Inf. Manag. 37, 284–296. doi: 10.1016/j.ijinfomgt.2017.03.004

[ref30] Fishbein M. Ajzen I. (1975). Intention and Behavior: An Introduction to Theory and Research. Addison-Wesley, Reading, MA.

[ref31] Fornell C. Larcker D. F. (1981). Evaluating structural equation models with unobservable variables and measurement error. J. Mark. Res. 18, 39–50. doi: 10.1177/002224378101800104

[ref32] Gefen D. (2000). E-commerce: the role of familiarity and trust. Omega 28, 725–737. doi: 10.1016/S0305-0483(00)00021-9

[ref33] Group A. (2014). The Internet of Things: The Future of Consumer Adoption ACQUITY GROUP.

[ref34] Gubbi J. Buyya R. Marusic S. Palaniswami M. (2013). Internet of things (IoT): a vision, architectural elements, and future directions. Futur. Gener. Comput. Syst. 29, 1645–1660. doi: 10.1016/j.future.2013.01.010

[ref35] Hair J. F. Hult G. T. M. Ringle C. Sarstedt M. (2016). A Primer on Partial Least Squares Structural Equation Modeling (PLS-SEM) Sage Publications.

[ref36] Hair J. F. Ringle C. M. Sarstedt M. (2011). PLS-SEM: indeed a silver bullet. J. Mark. Theory Pract. 19, 139–152. doi: 10.2753/MTP1069-6679190202

[ref37] Hair J. F. Ringle C. M. Sarstedt M. (2013). Partial least squares structural equation modeling: Rigorous applications, better results and higher acceptance.

[ref38] Han B. Wu Y. A. Windsor J. (2014). User’s adoption of free third-party security apps. J. Comput. Inf. Syst. 54, 77–86. doi: 10.1080/08874417.2014.11645706

[ref39] Haryoto K. S. (2015). The use of modified theory of acceptance and use of technology 2 to predict prospective users’ intention in adopting TV Streaming.

[ref40] Helkkula A. (2016). Consumers’ intentions to subscribe to music streaming services.

[ref41] Henseler J. Hubona G. Ray P. A. (2016). Using PLS path modeling in new technology research: Updated guidelines. Ind. Manag. Data Syst. 116, 2–20. doi: 10.1108/IMDS-09-2015-0382

[ref42] Hossain A. Quaresma R. Rahman H. (2019). Investigating factors influencing the physicians’ adoption of electronic health record (EHR) in healthcare system of Bangladesh: An empirical study. Int. J. Inf. Manag. 44, 76–87. doi: 10.1016/j.ijinfomgt.2018.09.016

[ref43] Hsu C.-L. Lin J. C.-C. (2016). An empirical examination of consumer adoption of internet of things services: network externalities and concern for information privacy perspectives. Comput. Hum. Behav. 62, 516–527. doi: 10.1016/j.chb.2016.04.023

[ref44] Huang C.-Y. Kao Y.-S. (2015). UTAUT2 based predictions of factors influencing the technology acceptance of phablets by DNP. *Math. Probl. Eng*, 2015.

[ref45] Ifinedo P. (2012). Technology Acceptance by Health Professionals in Canada: An Analysis with a Modified UTAUT Model. Paper Presented at the 2012 45th Hawaii International Conference on System Sciences.

[ref46] Järvinen J. Ohtonen R. Karjaluoto H. (2016). Consumer Acceptance and Use of Instagram. Paper Presented at the 2016 49th Hawaii International Conference on System Sciences (HICSS).

[ref47] Kalamatianou M. A. Malamateniou F. (2017). An extended UTAUT2 model for e-government project evaluation.

[ref48] Karahoca A. Karahoca D. Aksöz M. (2018). Examining intention to adopt to internet of things in healthcare technology products. Kybernetes 47, 742–770. doi: 10.1108/K-02-2017-0045

[ref49] Karaiskos D. C. Kourouthanassis P. Lantzouni P. Giaglis G. M. Georgiadis C. K. (2009). Understanding the Adoption of Mobile Data Services: Differences Among Mobile Portal and Mobile Internet Users. Paper Presented at the 2009 Eighth International Conference on Mobile Business.

[ref50] Khasawneh S. Jalghoum Y. Harfoushi O. Obiedat R. (2011). E-government program in Jordan: from inception to future plans. Int. J. Comput. Sci. Issues 8:568.

[ref51] Li C. Murad M. Shahzad F. Khan M. A. S. Ashraf S. F. Dogbe C. S. K. (2020). Entrepreneurial passion to entrepreneurial behavior: role of entrepreneurial alertness, entrepreneurial self-efficacy and proactive personality. Front. Psychol. 11:1611. doi: 10.3389/fpsyg.2020.01611 32973593 PMC7468520

[ref52] Limayem M. Hirt S. G. Cheung C. M. (2007). How habit limits the predictive power of intention: the case of information systems continuance. MIS Q. 31, 705–737. doi: 10.2307/25148817

[ref53] Lowry P. B. Gaskin J. Twyman N. Hammer B. Roberts T. (2012). Taking ‘fun and games’ seriously: proposing the hedonic-motivation system adoption model (HMSAM). J. Assoc. Inf. Syst. 14, 617–671.

[ref54] Mahfuz M. A. Khanam L. Hu W. (2020). Mobile banking services adoption: insight from brand name perspectives based on UTAUT2 model.

[ref55] Merhi M. Hone K. Tarhini A. (2019). A cross-cultural study of the intention to use mobile banking between Lebanese and British consumers: extending UTAUT2 with security, privacy and trust. Technol. Soc. 59:101151. doi: 10.1016/j.techsoc.2019.101151

[ref56] Moore G. C. Benbasat I. (1991). Development of an instrument to measure the perceptions of adopting an information technology innovation. Inf. Syst. Res. 2, 192–222. doi: 10.1287/isre.2.3.192

[ref57] Moura A. C. D. Gosling M. D. S. Christino J. M. M. Macedo S. B. (2017). Acceptance and use of technology by older adults for choosing a tour-ism destination: a study using UTAUT2. Rev. Bras. Pesqui. Turismo 11, 239–269. doi: 10.7784/rbtur.v11i2.1277

[ref58] Negahban A. Chung C.-H. (2014). Discovering determinants of users perception of mobile device functionality fit. Comput. Hum. Behav. 35, 75–84. doi: 10.1016/j.chb.2014.02.020

[ref59] Palau-Saumell R. Forgas-Coll S. Sánchez-García J. Robres E. (2019). User acceptance of mobile apps for restaurants: An expanded and extended UTAUT-2. Sustainability 11:1210. doi: 10.3390/su11041210

[ref60] Pavlou P. A. Tan Y.-H. Gefen D. (2003). Institutional Trust and Familiarity in Online Interorganizational Relationships. Paper Presented at the Proceedings of the European Conference on Information Systems (ICIS) Naples, Italy.

[ref61] Peljko Ž. Antončič J. A. (2022). Impacts of entrepreneurial openness and creativity on company growth. Front. Psychol. 13:860382. doi: 10.3389/fpsyg.2022.860382, PMID: 35846640 PMC9282161

[ref62] Qi L. Z. Ismail S. (2019). Factors Influencing Small and Medium Enterprises’ Behavior and Intention to Adopt Accounting Information System (AIS) Based Information Technology (IT). Paper Presented at the Proceedings of the 2019 2nd International Conference on E-Business, Information Management and Computer Science.

[ref63] Ramírez-Correa P. Rondán-Cataluña F. J. Arenas-Gaitán J. Martín-Velicia F. (2019). Analysing the acceptation of online games in mobile devices: An application of UTAUT2. J. Retail. Consum. Serv. 50, 85–93. doi: 10.1016/j.jretconser.2019.04.018

[ref64] Rogers E. M. (1995). Lessons for guidelines from the diffusion of innovations. Jt. Comm. J. Qual. Improv. 21, 324–328. doi: 10.1016/S1070-3241(16)30155-9 7581733

[ref65] Rogers E. M. (2010). Diffusion of Innovations Simon and Schuster.

[ref66] Sakarji S. R. Nor K. B. M. Razali M. M. Talib N. Ahmad N. Saferdin W. A. A. W. M. (2019). Investigating students acceptance of e-learning using technology acceptance model among DIPLOMA in office management and technology students at UITM MELAKA. J. Inf. 4, 13–26.

[ref67] Sarstedt M. Ringle C. M. Hair J. F. (2021). Partial Least Squares Structural Equation Modeling Handbook of Market Research Springer, 587–632.

[ref68] Sekaran U. Bougie R. (2011). Business Research Methods: A Skill-Building Approach: New York: McGraw-Hill.

[ref69] Sievers F. Reil H. Rimbeck M. Stumpf-Wollersheim J. Leyer M. (2021). Empowering employees in industrial organizations with IoT in their daily operations. Comput. Ind. 129:103445. doi: 10.1016/j.compind.2021.103445

[ref70] Slade E. L. Dwivedi Y. K. Piercy N. C. Williams M. D. (2015). Modeling consumers’ adoption intentions of remote mobile payments in the United Kingdom: extending UTAUT with innovativeness, risk, and trust. Psychol. Mark. 32, 860–873. doi: 10.1002/mar.20823

[ref71] Tarhini A. Alalwan A. A. Algharabat R. S. (2019). Factors influencing the adoption of online shopping in Lebanon: an empirical integration of unified theory of acceptance and use of technology2 and DeLone-McLean model of IS success. Int. J. Electron. Mark. Retail. 10, 368–388. doi: 10.1504/IJEMR.2019.104213

[ref72] Tarhini A. El-Masri M. Ali M. Serrano A. (2016). Extending the UTAUT model to understand the customers’ acceptance and use of internet banking in Lebanon: a structural equation modeling approach. Inf. Technol. People 29, 830–849. doi: 10.1108/ITP-02-2014-0034

[ref73] Tarhini A. Masadeh R. E. Al-Busaidi K. A. Mohammed A. B. Maqableh M. (2017). Factors influencing students’ adoption of e-learning: a structural equation modeling approach. J. Int. Educ. Bus. 10, 164–182. doi: 10.1108/JIEB-09-2016-0032

[ref74] Teo T. (2009). Modelling technology acceptance in education: a study of pre-service teachers. Comput. Educ. 52, 302–312. doi: 10.1016/j.compedu.2008.08.006

[ref75] Teo A.-C. Tan G. W.-H. Ooi K.-B. Hew T.-S. Yew K.-T. (2015). The effects of convenience and speed in m-payment. Ind. Manag. Data Syst. 115, 311–331. doi: 10.1108/IMDS-08-2014-0231

[ref76] Thompson R. L. Higgins C. A. Howell J. M. (1991). Personal computing: toward a conceptual model of utilization. MIS Q. 15, 125–143. doi: 10.2307/249443

[ref77] Thong J. Y. (1999). An integrated model of information systems adoption in small businesses. J. Manag. Inf. Syst. 15, 187–214. doi: 10.1080/07421222.1999.11518227

[ref78] Tseng T. H. Lin S. Wang Y.-S. Liu H.-X. (2019). Investigating teachers’ adoption of MOOCs: the perspective of UTAUT2. Interact. Learn. Environ., 635–650. doi: 10.1080/10494820.2019.1674888

[ref79] Tu M. (2018). An exploratory study of internet of things (IoT) adoption intention in logistics and supply chain management-a mixed research approach. Int. J. Logist. Manag. 29, 131–151. doi: 10.1108/IJLM-11-2016-0274

[ref80] Venkatesh V. Morris M. G. Davis G. B. Davis F. D. (2003). User acceptance of information technology: toward a unified view. MIS Q. 27, 425–478. doi: 10.2307/30036540

[ref81] Venkatesh V. Thong J. Y. Xu X. (2012). Consumer acceptance and use of information technology: extending the unified theory of acceptance and use of technology. MIS Q. 36, 157–178. doi: 10.2307/41410412

[ref82] Venkatesh V. Zhang X. (2010). Unified theory of acceptance and use of technology: US vs China. J. Glob. Inf. Technol. Manag. 13, 5–27. doi: 10.1080/1097198X.2010.10856507

[ref83] Zhu K. Kraemer K. Xu S. (2003). Electronic business adoption by European firms: a cross-country assessment of the facilitators and inhibitors. Eur. J. Inf. Syst. 12, 251–268. doi: 10.1057/palgrave.ejis.3000475

